# Visualization of Experience Sampling Method Data in Mental Health: Qualitative Study of the Physicians’ Perspective in Germany

**DOI:** 10.2196/72893

**Published:** 2025-12-22

**Authors:** Julia C C Schulte-Strathaus, Theresa Ikegwuonu, Anita Schick, Maria K Wolters, Lena de Thurah, Michal Hajdúk, Adam Kurilla, Inez Myin-Germeys, Glenn Kiekens, Jeroen D M Weermeijer, Joanne R Beames, Lotte Uyttebroek, Rafaël Bonnier, Iveta Nagyova, Dagmar Breznoščáková, Daniel Dančík, Koraima Sotomayor Enriquez, Islay Barne, Jessica Gugel, Ulrich Reininghaus, Michel Wensing, Charlotte Ullrich

**Affiliations:** 1 Department of Public Mental Health, Medical Faculty Mannheim, Central Institute of Mental Health, Heidelberg University, J5, Mannheim, 68159, Germany, 062117033734; 2 Centre for Medical Informatics, USHER Institute, University of Edinburgh, Edinburgh, United Kingdom; 3 School of Informatics, University of Edinburgh, Edinburgh, United Kingdom; 4 Department of Neuroscience, Centre for Contextual Psychiatry, KU Leuven, Leuven, Belgium; 5 Department of Psychology, Faculty of Arts, Comenius University Bratislava, Bratislava, Slovakia; 6 Department of Medical and Clinical Psychology, Tilburg University, Tilburg, The Netherlands; 7 Faculty of Psychology and Educational Sciences, KU Leuven, Leuven, Belgium; 8 Department of Social and Behavioural Medicine, Faculty of Medicine, University of Pavol Jozef Šafárik, Košice, Slovakia; 9 Department of Psychiatry, Faculty of Medicine, Comenius University Bratislava, Bratislava, Slovakia; 10 Department of Clinical Psychology, University of Edinburgh, Edinburgh, United Kingdom; 11 Centre for Epidemiology and Public Health, Health Service and Population Research Department, Institute of Psychiatry, Psychology & Neuroscience, King's College London, London, United Kingdom; 12 Partner site Mannheim‑Heidelberg‑Ulm, German Center for Mental Health (DZPG), Mannheim, Germany; 13 Medical Faculty, Heidelberg University, Heidelberg, Germany; 14 Department of General Practice and Health Services Research, University Hospital Heidelberg, Heidelberg, Germany

**Keywords:** data visualization, experience sampling method, mHealth, mobile health, personalized care, qualitative research

## Abstract

**Background:**

Although the integration of self-monitored patient data into mental health care offers potential for advancing personalized approaches, its application in clinical practice remains largely underexplored. Capturing individuals’ mental health outside the therapy room using experience sampling methodology (ESM) may bridge this gap by supporting shared decision-making and personalized interventions.

**Objective:**

This qualitative study investigated the perspectives of German mental health professionals regarding prototypes of ESM data visualizations designed for integration into a digital mental health tool.

**Methods:**

Semistructured interviews were conducted with clinicians on their perceptions of such visualizations in routine care.

**Results:**

Using reflexive thematic analysis, 3 key findings emerged (1) ESM and ESM data visualizations were seen as valuable tools for enhancing patient motivation and engagement over the course of treatment; (2) simplicity and clarity of visual formats, particularly line graphs, were preferred for usability; and (3) practical concerns, such as workflow integration challenges centered on time constraints (psychotherapy session duration 50 min) and need for patient psychoeducation materials, influenced perceived utility. Challenges, including the risk of cognitive overload from dense data representations (eg, ESM mood-in-context visualizations), were raised.

**Conclusions:**

These findings underline the importance of designing digital tools that align with clinical needs while addressing potential barriers to implementation by exploring the opportunities and challenges associated with ESM visualizations.

## Introduction

Smartphones have become valuable tools in health contexts, enabling the collection of real-time, scalable health data that track behaviors such as sleep, physical activity, and symptoms. These devices can gather both “passive” (eg, step count, heart rate) and “active” data (eg, survey data), providing valuable insights into an individual’s health over time [1,2]. As a result, mobile health (mHealth) tools have gained significant attention for their potential to offer a more comprehensive understanding of both physical and mental health [3]. mHealth tools, such as those used for monitoring conditions like depression [4], substance use disorders [5], and schizophrenia [6], show increasing promise in improving patient care. A key method within this field is the experience sampling method (ESM), which deploys smartphone prompts or ecological triggers throughout the day to facilitate real-time data collection about individuals’ thoughts, feelings, and behaviors in their natural environments [7,8] and the delivery of interventions [9] directly to the user. Unlike retrospective recalls, ESM captures momentary experiences as they occur, providing rich temporal and contextual intensive longitudinal data about symptom patterns, triggers, and daily life influences. Despite its potential, however, data collected with ESM are rarely shared with patients or integrated into routine clinical practice. This gap may stem from the ongoing challenge of transforming raw ESM data into meaningful, actionable visualizations that clinicians can use effectively in patient care [4].

The necessity to improve data visualizations in health care is becoming increasingly evident [10-12]. Research suggests that data visualizations can improve patient engagement and compliance by helping them better understand important trends in their health data by enhancing the ability of both clinicians and patients to explore, interpret, and discuss complex data [8,13,14]. Visual tools for personal health care data can help patients improve their health literacy, support informed decision-making, and speed up the comprehension of complex information [15,16]. By recognizing patterns over time, users may gain new insights into their own health signals and triggers, empowering them to manage their health proactively [17]. Furthermore, effective visualizations can build trust and encourage patients to share their smartphone data, as they offer a transparent view of how their information is utilized in their care [18]. Simple line graphs are particularly effective for depicting temporal changes, as they make trends and patterns easily discernible [19,20].

While the benefits of visualizations are noteworthy, there are potential adverse effects that warrant consideration. First, overly complex visualizations can pose challenges for both clinicians and patients and confuse or overwhelm patients, particularly those with lower health literacy or cognitive impairments [21]. This may lead to disengagement or frustration, undermining the intended trust-building effect. Second, if patients perceive the visualizations as reductive, they may feel that their experiences are being trivialized, which could further erode confidence in the tool or the clinician’s understanding of their condition [22]. Third, patients may misinterpret visualized data without adequate guidance from clinicians, potentially leading to unfounded concerns or inaccurate self-assessments, which might exacerbate anxiety [4].

Despite promising evidence for ESM-based monitoring and feedback, clinical uptake remains limited, with barriers spanning clinician time and workflow fit, cognitive load of feedback, and governance or technical integration (eg, dashboards integrated with routine sessions). The clinician perspective on when and how ESM visualizations are usable, useful, and safe in routine psychotherapy is currently underexplored, particularly in Germany. This study aims to explore German mental health professionals’ perceptions of ESM data visualizations, focusing specifically on preferences, perceived barriers, and integration considerations for clinical practice.

## Methods

### Study Design

To explore how clinicians within the mental health sector perceive and utilize visualizations of ESM data, a qualitative study design using semistructured interviews was chosen. This qualitative study forms part of the Implementing Mobile Mental Health Recording Strategy for Europe (IMMERSE) project, dedicated to advancing person-centered mental health care across 4 countries in Europe (Belgium, Germany, Slovakia, and the United Kingdom) through the development of a Digital Mobile Mental Health (DMMH) tool [23,24]. For this study, the screenshots of 3 prototypes (line graph, mood in context, pie chart) were selected based on preliminary stakeholder interviews within the IMMERSE consortium, representing common temporal, contextual, and proportional data representation approaches in existing ESM platforms. The visualizations are to be integrated into an mHealth tool in a later study phase [23,24].

### Study Population, Recruitment, and Sampling

To be eligible for participation in this study, clinicians needed to be part of the IMMERSE study in Germany, which took place at 2 psychiatric hospitals: the Central Institute of Mental Health (Mannheim, Germany) and the Psychiatric Centre Nordbaden (Wiesloch, Germany). Both centers have multiple departments working in interdisciplinary teams. Purposeful sampling was employed to ensure diversity in clinical backgrounds (ie, psychologists and psychotherapists from different wards) and experience levels (senior vs in training). Recruitment continued until thematic saturation was reached.

### Data Collection

Semistructured guide–based interviews used in this study were conducted by 1 female interviewer (JCCSS), with a background in clinical psychology and psychotherapy. Participants were either interviewed face-to-face at their workplace or via video call according to the participants’ preferences. Based on predefined research questions, an interdisciplinary research team from all IMMERSE participating field sites developed the semistructured interview guide in the respective languages for all countries and the 4 different stakeholder groups (clinicians, patients, friends and family members of patients, admins or information technology). Interviews were recorded using audio recorders.

The interview guide for clinicians in Multimedia Appendix 1 comprised 27 questions focusing on (1) prior experiences with digital health tools; (2) perspectives on app usability and patient engagement, potential barriers; (3) feedback on the DMMH item prototype; and (4) the expected impact of app usage on therapy outcomes. A total of 3 prototypes (Figures S1-S3 in Multimedia Appendix 2) of the visualized longitudinal patient data were shown and explained to participating clinicians for feedback. In addition, vignettes with clinician case examples were presented and discussed in Multimedia Appendix 1.

### Data Analysis

All interviews in this study were transcribed verbatim by student assistants in German and then coded by JCCSS in English, who maintained analytic memos. The interview data were analyzed using reflexive thematic analysis, following the 6-step framework outlined by Braun and Clarke [25,26]: familiarization with the data, generating initial codes, searching, reviewing, defining and labeling patterns of results, and producing the final report. Inductive coding provided a structured method for identifying and interpreting patterns within the data [27]. This approach ensured a thorough and nuanced understanding of participants’ perspectives, balancing the exploration of preidentified topics with the discovery of novel insights. The analysis was conducted using the MAXQDA software [28], which supported the systematic organization and management of the data. Coding and theme development were performed iteratively, with JCCSS and TI independently reviewing the data to enhance reliability. Discrepancies in coding were resolved through regular consensus discussions between JCCSS and CU, fostering a reflexive analytical process [29]. Themes were discussed by all the authors and compared to interview transcriptions to check their encompassment of the data. Included quotes have been translated into English by JCCSS with due diligence and are cited with the indication of participant number and transcript position. For clarity, we present the findings through 3 lenses: (1) clinicians’ perceptions, (2) their views on patients’ perceptions, and (3) their views on future use cases. Each lens contains material from 1 or more themes.

### Open Science

The following paper was preregistered on January 29, 2024 (osf.io/mjqd7). There were no deviations from the preregistered protocol.

### Ethical Considerations

Ethical approval was granted on September 24, 2021, by the Ethics Committee II of the University of Heidelberg at the Medical Faculty Mannheim (approval reference 633‐21). The source of funding for this project is the EU-Horizon 2020 program under the IMMERSE project (European Union, Horizon 2020 [H2020 – 945263]). All participants signed an informed consent form prior to participation. Participation was voluntary, and participants could withdraw from the study at any time without negative consequences. The data were collected and stored in pseudonymized form on secure institutional servers. Only authorized study personnel had access to the data, and no personal identifying information is reported in the results. Participants received €10 (~11.65 US $) as compensation for their time.

## Results

### Participant Characteristics and Overview

Between January and June 2022, a total of 14 clinicians were interviewed, primarily in a face-to-face setting. Most clinicians were female psychologists (education: master’s or above) in advanced postgraduate education to become licensed psychotherapists and differed in terms of focalized versus comprehensive fields (Table 1). Interviews were between 40 and 60 min with a mean duration of 50 min (SD 15 min).

A total of 3 themes structured our analysis: (1) motivation and engagement, (2) simplicity and clarity, and (3) workflow fit and cognitive load. For presentation, we organize the findings into 3 sections. These reporting sections are not distinct themes themselves but lenses through which we present clinicians’ accounts; within each, we indicate how they relate back to the 3 themes.

**Table 1.** Sociodemographic characteristics of mental health professionals.

Participant characteristics	Frequency
Assessment site, n (%)	
PCN^a^	2 (14.3)
CIMH^b^	12 (85.7)
Age (y), mean (SD)	36.9 (10.9)
Gender, n (%)	
Female	10 (71.4)
Male	4 (28.6)
Profession, n (%)	
PPiA^c^	9 (64.3)
Doctor of medicine	4 (28.6)
Social work	1 (7.1)

a PCN: Psychiatric Centre Nordbaden.

b CIMH: Central Institute of Mental Health.

c PPiA: psychologists holding a Master Title of Clinical and Cognitive Psychology in practical training to become a licensed psychotherapist within the German clinical training system (German: *Psychologische Psychotherapeut:in in Ausbildung*).

### Clinicians’ Perceptions

When asked for first impressions of the prototypes, clinicians shared insights and suggestions for enhancing the design and functionality of the ESM data visualization prototypes.

First, in fast-paced work environments, where decisions often need to be made rapidly, the intuitiveness of a visual tool is crucial. *Complexity* in visualizations can hinder quick comprehension, which is a critical consideration in a clinical work setting. One clinician stated:

Okay – well, I actually needed a second to understand how the diagram is structured. I have to say, it’s not exactly intuitive.

[Z_01_Pos.95]

This quote illustrates that while visualizations may convey rich data, they can also pose an initial barrier to understanding. The clinician’s experience suggests that Figure S2 in Multimedia Appendix 2 may require a moment of adjustment or interpretation, which could be a drawback in situations where time is limited. This preference hierarchy reflects an efficiency-accuracy tradeoff common in clinical decision-making. While some clinicians valued the contextual richness of the mood-activity mapping of Figure S2 in Multimedia Appendix 2 (see the *Clinicians View on Patients’ Perceptions* section), others reported they would default to the line graph in Figure S1 in Multimedia Appendix 2. Second, clinicians provided feedback on the *design and layout* of the visualizations, suggesting specific changes that could improve their usability and effectiveness, for example, by rotating images and using lists (Z_01_Pos.97).

Moreover, currently, analog forms of patient data were perceived as cumbersome, and interest in digitally tracking currently analog data through more user-friendly means, such as digital visualizations, was expressed as illustrated within 1 interview.

There are these mood-tension curves from DBT [Dialectical Behavioural Therapy], I haven't used them yet because I always find them a bit unhandy, but I've often thought yes, it would be cool if you could track something like that.

[P_02_Pos.80]

This reflects a broader concern regarding the accessibility of information. By adjusting the layout to make it more intuitive or easier to interpret, visualizations could perhaps better support interactions with patients and improve the clarity of the data being communicated. This aligns with the need for striking a *balance between providing detailed information and maintaining clarity* in visualizations, especially over the course of different sessions. Clinicians acknowledged that while adding more categories or data points can increase the richness of data in a visualization, this does not necessarily detract from its usefulness. One clinician reflected:

No, I don’t find the amount of categories particularly problematic. A pie chart is something that everyone should be able to interpret in some way. And – yeah, I don’t think it’s an issue to include several categories. Of course, the more categories there are, the more complex it becomes. You could consider combining some of them, but in principle, I don’t find it problematic.

[Z_12_Pos.241]

This underscores the importance of careful design in creating visualizations that are both informative and accessible, ensuring that they serve as effective tools in the fast-paced and demanding environment of clinical practice. By understanding and addressing the fundamental tension between comprehensive data representation and cognitive efficiency, developers may create visual tools that better meet the needs of clinicians and support more efficient and accurate decision-making processes.

### Clinicians' View on Patients’ Perceptions

When asked about their patients’ perspectives, clinicians highlighted the possibility of enhanced motivation, a desire for personalization, and worries regarding cognitive load. Clinicians stressed that the true value of the visualization lies in the patient’s ability to comprehend and engage with it, perhaps highlighting a gap between theoretical appreciation and practical implementation.

First, clinicians noted the powerful role that data visualizations could play in *changing and enhancing patient motivation*. Visual feedback could be used effectively in therapy to reinforce positive behaviors and encourage patients to take more proactive roles in their treatment. One clinician described:

I find that very interesting. I think it’s something you can discuss really well with patients, like saying, ‘Look at this, it seems like being active is really beneficial. Why not try doing that more?’ I could really use something like that.

[P_02_Pos.68]

In making abstract concepts such as mood or activity levels more tangible, ESM data visualizations may contribute to a *patient’s sense of self-management* and control over their mental health (data). Clinicians pointed out the importance of giving patients access to the visualizations and viewed this approach as a way to strengthen the patient’s intrinsic motivation, which is crucial for long-term engagement and success in therapy (eg, Z_06_Pos.57‐59). They advocated for giving patients access to their own data and emphasized the importance of empowering patients to take an active role in their own care and decision-making processes as seen in the following interview quote:

I can only make use of the data if the patient wants to. I also think that this is the patient’s true motivation – that they feel like they have control over themselves and can engage in self-management and self-control. That’s the most powerful motivational argument in treatment. It’s something that shouldn’t be dismissed lightly, so it’s important to also give the data back to the patient.

[Z_06_Pos.59]

Second, clinicians underlined the importance of flexibility in ESM visualizations, enabling patients to track specific behaviors or concerns that are pertinent to their personal experiences. Such customization may make the visualizations more “relevant” and actionable, thereby potentially enhancing their value, as 1 clinician described:

Well, it depends on what the patient is doing. For certain disorders, definitely yes. But maybe it would only be useful if one could also enter activities themselves or note what they’re preoccupied with. For example, if there were an option to add things like ‘worrying’ or ‘being preoccupied with my appearance and body’, something like that. If you could add these kinds of things, it might also help capture problematic behaviours.

[Z_09_Pos.60]

Third, clinicians expressed concerns about the *risk of overwhelming patients* with too many options and complex interfaces in visual data tools. While the ability to customize and compare various data points can be beneficial for clinical practice, it may also be burdensome for some patients, as 1 clinician explained:

Yes… it definitely needs to be well-selected. In the end, it could easily become overwhelming, especially for highly burdened patients who may also have cognitive impairments. From my perspective, I think, ‘Oh, I want to be able to compare everything with each other, and I want a lot of options to customise things myself.’ For example, if I suddenly have the idea to compare mood with the sleep from the previous night, I think it would be incredibly helpful to always have the possibility to visualise all my ideas. But in contrast, for patients, it might be overwhelming if they are given the option to adjust everything themselves, as this would likely be too much for them to handle.

[Z_01_Pos.105]

This quote reflects a tension between the clinician’s desire for detailed and customizable tools and the assumed potential for these features to become a source of stress for patients.

### Clinicians' View on Future Use of ESM Data Visualizations in Routine Care

When asked about future use cases, clinicians highlighted the practical value of ESM data visualizations in enhancing *patient communication*. First, emphasis was placed on the importance of involving the patient in the process of understanding and interpreting their own data visualization. This understanding can only be achieved through dialogue and collaboration between the clinician and the patient, with clinicians imagining discovering new insights about the patient’s perspective and experiences during data-driven discussions. Overall, the following quote underscores a general interest in shifting toward patient empowerment and collaboration, highlighting the value of involving patients in understanding and utilizing their own data:

So, when I look at this, I can certainly glean some insights, but it only truly becomes valuable when the patient can also see it. And I can only really develop that during a conversation with them – figuring out how much they can perceive at the moment, whether they can follow me. Often, entirely new insights emerge for me that I wasn’t aware of before, only when the patient explains them to me. I used to be much more paternalistic, especially when I started out around 30 years ago. I thought, ‘Ah, I have it all figured out.’ But I don’t think that way anymore. I can only make sense of the data if the patient is willing.

[Z_06_Pos.57-59]

Clinicians consistently highlighted the utility of graphical representations in their clinical practice: line graphs in particular were seen as essential for making abstract data—such as mood fluctuations—more concrete and accessible to patients. One clinician pointed out the value of graphical representation of ESM as a *communication tool*:

Yeah, somehow with numerical averages – hm, I’m not sure. Probably, especially for mood items or affect-related aspects, graphical representations would be best. On one hand, it’s information for me, for example, for my ward rounds or something similar. But on the other hand, I would also want to bring this into conversations with patients. And then it’s incredibly helpful to show them something like, ‘Look at this line graph, see how your afternoons are always very high.’ It’s much more illustrative that way, and I believe it makes it easier to discuss and work with psychotherapeutically.

[Z_01_Pos.89]

This quote underscores how line graphs may facilitate clearer communication and help patients better understand their own emotional patterns, in turn perhaps enhancing the interaction quality.

A second notable aspect accentuating utility in practice was a desire for visualizations to *integrate mood data with other relevant variables*, such as activities, specific symptoms, mood, sleep, and therapy goals. Clinicians emphasized that these correlations may be crucial for providing a comprehensive understanding of the patient’s condition. One participant remarked:

It is also very meaningful to not only measure mood itself but also to consider its connection, in this case, with activities or, for example, with various symptoms – like warning signs in connection with mood or whatever else might be relevant.

[Z_01_Pos.97]

Such integration may facilitate clinicians in identifying patterns and triggers, making it easier to address specific issues during therapy. Moreover, visualizations that allow clinicians to *correlate mood data with specific issues*, such as substance use or relapse risks, were particularly valued. Such tools may enable clinicians to address complex therapeutic concepts (eg, self-medication hypothesis) in a more tangible way:

When thinking about functionality, one might work with a patient to address, for example, their avoidance of boredom or feelings of emptiness, or whatever the patient individually seeks to achieve through substance use. Being able to correlate this could also help in working through a relapse. When considering relapse processing, this could be a useful approach.

[P_01_Pos.62]

Clinicians also showed a strong interest in the visualization prototype that illustrated the *relationship between activities and mood*. These visualizations were considered valuable for guiding patients toward behaviors that enhance mood and overall well-being. As one clinician articulated:

This means that, by using something like this, I could work with the patient to identify, for example, that positive activities, being productive, and engaging in social contact are more likely associated with a good mood. In contrast, if the patient spends time just watching TV or doing nothing – what might fall under the category of “nothing” – we might see more instances of low mood.

[Z_08_Pos.58]

Third, the use of visualizations for *goal setting and tracking progress* toward therapy goals reflected another notable use case for clinicians. Clinicians saw value in using these tools to monitor how well patients are meeting their therapeutic objectives as visualizations may provide patients with a clear sense of their progress, which can be motivating and reinforcing.

What I find very meaningful is working with patients to create an individualised therapy goal plan and then regularly checking with them to see how far they’ve progressed regarding the goals that were formulated together, I find that very meaningful. Of course, activities are important, and correlating them with mood seems effective, at least. Addiction treatment often involves the topic of identifying meaningful activities.

[P_01_Pos.60]

In sum, this highlights the role that ESM data visualizations could play in enhancing clinical practice in the future. Clinicians see these visualizations as not just informative but perhaps valuable for patient communication, personalized treatment, and goal tracking.

## Discussion

### Overview

This study explored German clinicians’ perspectives on the visualization of patient-reported ESM data by using 3 prototypes of (1) line graph; (2) mood in context of activities; and (3) pie chart, with a specific focus on its potential integration into routine clinical care. The findings indicate that clinicians perceive ESM visualizations as valuable for supporting patient self-management and enhancing therapeutic dialogue in sessions. However, preferences for specific visualization formats varied, and concerns about complexity and usability emerged, particularly in time-constrained clinical environments.

### Visualizing Real-Time Moods

Clinicians in this study generally acknowledged the utility of ESM visualizations in providing a dynamic view of patients’ mental health, particularly in tracking mood fluctuations and activity levels over time. This aligns with existing literature on the benefits of ESM in mental health care, which highlights the potential of real-time data to inform treatment decisions and improve outcomes [30,31]. However, while clinicians found simple line graphs helpful for visualizing temporal changes in mood, they were more skeptical about the usefulness of more complex formats, which were sometimes viewed as overwhelming or difficult to interpret. This concern is consistent with previous research suggesting that the design of visual tools in health care must strike a balance between providing sufficient detail and being easily interpretable [4]. In particular, visualizations with too many data categories or excessive granularity may increase cognitive load, potentially limiting their practical use in busy clinical settings [13,32]. Therefore, the findings tentatively suggest that while visualizing ESM data holds promise for improving clinical practice, careful attention to the design and simplicity of such tools is necessary to ensure their usability. To address this flexibility-complexity paradox, specific design strategies could include progressive disclosure interfaces that allow clinicians to start with simplified views and drill down into detail as needed, or adaptive dashboards that learn from user interaction patterns to present most relevant information first.

Several studies have demonstrated the potential of ESM to offer clinicians a more nuanced understanding of patients’ daily mental health experiences [7,17,19]. In line with these studies, clinicians in this study emphasized that ESM visualizations could facilitate more personalized interventions, particularly by simplifying the process of identifying patterns of behavior that correlate with mood changes.

However, this study also highlights a tension between the perceived utility of these tools and their practical application. For instance, echoing findings from a US hybrid telehealth clinic study [4], visualizations of ESM data functioned as conversation starters and could be motivational, yet in-session use varied; perceived accuracy hinged on patient engagement, and a digital navigator often steered plot selection. Moreover, similar to previous findings [33,34], some clinicians expressed concerns about the time required to interpret complex visualizations, particularly when working with patients who may have cognitive impairments or limited data literacy. Therefore, while ESM visualizations are seen as valuable, their successful integration into routine care may depend on how effectively they can be simplified and tailored to meet the needs of both clinicians and patients. This is largely contingent on how a clinician may want to integrate the visualizations in daily practice.

### Patient Involvement and Empowerment

Clinicians frequently mentioned that visualizing patient data could enhance patients’ sense of autonomy and self-management, which is consistent with research on patient-centered care and motivational strategies in mental health treatment [35]. Presenting patients with a visual representation of their mental health patterns may foster a greater sense of control, potentially improving engagement and adherence to treatment plans, but also as an informative tool to capture perhaps non-pathological fluctuations in daily life (ie, normalizing ups and downs) [13,20,31].

Nonetheless, clinicians cautioned that the effectiveness of such tools depends on how well patients can understand and relate to the visualized data. As highlighted previously [19], patients’ ability to engage with digital health tools is highly variable and may be significantly diminished under conditions of heightened distress or cognitive burden. ESM data visualizations might increase patient anxiety about mood fluctuations or lead to over-interpretation of normal variations. Thus, while ESM visualizations have the potential to empower patients, their implementation should be accompanied by patient education components and clear guidance on data interpretation limits.

### Design and Customization of Visual Tools

The findings also highlight the importance of customization in the design of ESM visualizations. Several clinicians expressed a desire for more flexible tools that allow them to adjust the visualizations to reflect specific patient behaviors or symptoms, for example, a digital navigator that steers plot selection [4]. This aligns with the growing trend toward personalized health technologies, which emphasize the need for adaptable tools that cater to individual differences in patient experience [36]. Customization options, such as the ability to correlate mood with specific activities or life events (Figure S2 in Multimedia Appendix 2), were seen as useful for maximizing the clinical utility of ESM data. However, there are also potential drawbacks to customization. Clinicians noted that overly flexible or complex visualization tools could become burdensome, particularly for patients who might be overwhelmed by too many options or unfamiliarity with data’s interpretation, as demonstrated in the literature [32].

The findings suggest several practical implications for the use of ESM visualizations in mental health care. First, simplicity and clarity in design are essential for ensuring that these tools can be effectively integrated into routine clinical practice [11,14,15,20,32]. As digital health tools become more widespread, it will be important to examine how ESM visualizations can be adapted for use in various mental health care contexts, including outpatient care, community mental health services, and telemedicine. Further research should also consider the integration of these tools within electronic health records, ensuring that they are compatible with existing clinical workflows and data systems [34]. Moreover, visualizations must be intuitive enough to be interpreted quickly, particularly in time-constrained settings. Second, involving patients in the interpretation of their ESM data may enhance their engagement and sense of ownership over their treatment, which could improve therapeutic outcomes [35,37]. Third, as with the implementation of other tools in health care [36], clinicians will likely require some training and support. As such, any implementation of ESM visualizations should be accompanied by comprehensive training that ensures clinicians feel confident in their use.

### Methodological Considerations

The qualitative design of this study, utilizing semistructured interviews, provides an in-depth understanding of clinicians’ perspectives on visualizing ESM data within mental health care. Purposeful sampling ensured diversity in clinician expertise and backgrounds, enhancing the applicability of the findings across different levels of clinical experience. However, the sample size, while diverse, remains limited to 2 clinical institutions in Germany, which may affect the generalizability of the findings to other national or international contexts. This composition likely shaped how participants appraised the visualizations. First, the early-career majority (Table 1; mean age 36.9 y, SD 10.9) may have amplified preferences for simple, didactic displays that support psychoeducation and shared decision-making, and heightened sensitivity to cognitive load, a pattern reflected in comments about needing “a second to understand how the diagram is structured,” and the tendency to default to line graphs for rapid sense-making in time-pressed settings. Moreover, while all clinicians reported general mobile health familiarity (eg, step counters, cycle tracking), direct experience with ESM-based feedback was limited. This likely contributed to calls for brief primers and to concerns about overwhelm with dense, context-rich views. Illustratively, participants contrasted the appeal of digitally tracking familiar analog tools (eg, mood-tension curves in dialectical behavioral therapy) with reservations about complex screenshots.

 The use of visual prototypes and vignettes strengthened the study by making abstract concepts more concrete, thus facilitating more informed and practical reflection. A notable limitation was our use of static screenshots rather than interactive prototypes. This approach likely influenced participant feedback, as static visualizations cannot convey the full user experience of filtering, zooming, or dynamic data exploration that would characterize the actual clinical implementation. This study focused on the view of health care providers. To understand how patients perceive and interact with these visualizations and view their use within consultation, future research should include in-depth interviews with patients and direct observations of consultations using ESM data-visualizations.

In general, future research could benefit from including a wider range of visual formats or using codesign approaches [36], where clinicians and patients collaboratively develop visualization tools that better suit their needs.

### Conclusions

This qualitative study investigated the use of ESM data visualization in mental health care from the perspective of clinicians in Germany, from which we draw 3 main conclusions. First, clinicians expressed a strong desire for comprehensive data visualization options that enable the comparison and individualization of patient data. Second, concerns were raised about the potential for overwhelming patients, particularly those with cognitive limitations, due to excessive customization options. Third, and perhaps most importantly, the study observed a shift toward personalized care, with an emphasis on empowering patients to interpret their own data. Clinicians noted the motivational benefits of involving patients in self-management, stressing the importance of tools that enhance patient engagement. Clinicians valued simple-by-default, workflow-aligned ESM visualizations. We recommend (1) implementing summary-first line-trend defaults with literacy aids, (2) embedding time-bounded review prompts (≤5 min) suitable for 50-minute sessions aligned with German psychotherapy billing codes; and (3) evaluating these design choices in future studies. In conclusion, the study advocates for the development of data visualization tools that prioritize the needs and preferences of patients while maintaining their utility for clinicians.

## Supplementary material

10.2196/72893 Multimedia Appendix 1 Interview guide.

10.2196/72893 Multimedia Appendix 2 Prototype ESM Data Visualizations Shown to Clinicians as Discussion Stimuli.

## Abbreviations

ESM: experience sampling method
IMMERSE: Implementing Mobile Mental Health Recording Strategy for Europe
mHealth: mobile health
