# Supplementary material for: Visualization of Experience Sampling Method Data in Mental Health: Qualitative Study of the Physicians’ Perspective in Germany
Source: J Med Internet Res. 2025 Dec 22;27:e72893. doi: 10.2196/72893 (PMC12721489; doi:10.2196/72893)
Supplement: Multimedia Appendix 1 [file jmir-v27-e72893-s001.docx]

Supplementary Material

**Interview guide**

Introduction and practical information

My name is ______ , I am working as a researcher with the IMMERSE study team, and I will be conducting this interview today.

First of all, thank you for taking the time to talk to me. You sharing your views and experience is very valuable for us to understand the clinical context in which DMMH will be used and how we can improve the DMMH app. Please remember there are no ‘right’ or ‘wrong’ answers, we would simply like to hear more about your experiences and views in relation to using mental health apps.

In this interview, we will discuss different themes. We will focus on your previous experience with and potential future use of mental health apps, your opinion on a prototype of the DMMH app, how it might be used in the context of clinical care, and the impact it may have on you(r therapy) and patients’ daily life.

This interview will be audio recorded. The recordings will be saved using an anonymized study ID and transcribed into text by a team member, which can then be used for analysis.

The interview will take approximately 45 - 60 minutes. If you would like a break at any point, please let me know.

Are you happy for me to record this interview? This is so we remember all the details of our discussion. (If yes, **put on voice recorder**.)

Do you have any questions at this point? If not, I will start the interview.

You have been invited to take part in this interview as you responded to our invitation (ad/call for participants).

We are interested in speaking to a wide range of people, so we ask participants a few questions about their background.

Age:

Gender:

Ethnicity:

Employment status:

Education:

Area of residence/city/health board:

1. To begin with, can you tell me a bit more about **your role**?

*Further probes: What is your caseload like? How often do you see patients? How much time do you get to prepare for each patient?*

I would like to learn a bit more about your **previous experience with mental health apps**.

1. As far as you are aware, have your patients ever used any app for their mental health or other self-monitoring apps?

If yes, to what extent have they brought these data into the consultation?

1. Have you personally ever used any self-monitoring apps? *Further probes: Why (not)? If yes, which ones did you use? Are you still using them? If not, why did you stop?)*

I would like to show you a **prototype of the DMMH app** and talk about your views on using it. [Interviewer will explain about how the app works/its key aims/show the screen recording video].

I would like to know a bit more about your views of the app and your opinion on the questions that your patients would complete.

4. What concerns do you have using a self-monitoring app such as DMMH? What do you like/dislike?

5. What do you think about patients paying attention to their feelings multiple times a day and recording these via the app?

6. What do you think of the questions within the app? Do you find them relevant?

7.   Is there anything else that you would like patients to monitor in relation to their mental health? Anything else you would like to see included in the app? [e.g., physical activity, food, sleep]

8.     If you could **make any changes to** the app (e.g. adding questions or answer options), what would you do?

[…] We are now going to talk about how you like to learn about using new technologies and procedures, and what kind of training you would prefer. First, here is an example (vignette).

| Pamela is a clinician who specialises in mental health. She was given a 37-page manual explaining how to use the DMMH app, but she did not have the time to read the manual. This led to a lack of knowledge of how to use the app properly. When discussing this with the researchers, they worked out that Pamela would be able to attend a hands-on training session with the researchers. This helped her a lot to use the app more effectively. |
| --- |

9. How do you like to learn about new apps? What kind of **training/support** would help you to integrate DMMH into clinical practice? *[If interviewee does not come up with options, mention example videos, written instruction manuals, online training, learning collaborations].*

10.   How much **time** would you find acceptable for training?

11.   Would you prefer to work through the training material independently (at your own pace and at any time) or would you like fixed times to attend training (several sessions/one session only, what would fit into your schedule)?

[…] I would like to talk about **notifications**/alerts now. I’ve got an example (vignette) to illustrate this.

| Thomas works as a nurse, and he is mostly on night shifts. While doing his self-monitoring period using DMMH, the app would send him regular alerts throughout the day. This would disturb his sleep, and he would feel guilty (and a little bit annoyed) about not completing the questions at each notification.  The app does have a customisation option, so together with his clinician, Thomas was able to change the time he would get alerted to be more suitable for his work pattern. |
| --- |

12.   How do you feel about using alerts and reminders on your phone personally?

13.  How much **time** would you find acceptable for patients to complete the questions? (per beep/reminder, per day)

14.  Do you sometimes experience any **technical problems** while using your phone/tablet/work PC (e.g. connectivity issues, screen freeze, glitches)? Could this be an issue for using the DMMH app with patients in therapy?

[…] We’re about halfway through the interview. I am now going to ask you some questions on the **possibilities DMMH offers in relation to data feedback/integration of the app into clinical care** and the impact that using the DMMH app may have on your patients, their therapy and/or yourself. The idea is that you would meet up with your patients after their self-monitoring period to discuss their results. First, here is an example of how the DMMH app could work in practice.

| Mark took out early retirement due to his health. He used the DMMH app to get an insight into whether his current treatment is working. It became very clear to him that a major factor for his mental health is stress. He found out more about triggers that make him feel stressed. He said that “using the app showed me that I am not good at coping with stress.” He was able to address this with his clinician and they discussed various ways that can help him cope when he feels very stressed. These included behavioural interventions such as doing more physical activity and spending time outdoors. |
| --- |

15.  What do you think about discussing information captured by DMMH with your patients? How would you integrate it into clinical care? Any challenges?

16.  How much **time** would you need to prepare the (intervention / feedback) sessions based on information from the app?

17.  How do you think that DMMH might affect patients’ ongoing therapy, and their role in therapy? *[e.g., feeling more motivated/engaged in decisions about their clinical care].*

I would like to know a bit more about the **impact that using the DMMH app may have on clinical care.**

18.  After using/seeing a prototype of the DMMH app, will it solve any challenges you are currently experiencing within clinical care? If yes, can you give an example? If no, why not?

 We have come to the last part of the interview. I would like to talk very briefly about possible future use of digital health apps and any suggestions for improvement.

19.  Would you like to use DMMH (or a similar self-monitoring app) with patients in the future? Why (not)?

20.  Is there anything you would change about the DMMH app? What and why?

21.  Is there anything else you would like to add? Do you have any questions?

**Thank you again for your time.** We really appreciate you participating in this study. If you have any further questions regarding the study, you can always contact us on our email address or phone number.
