# Supplementary material for: Visualization of Experience Sampling Method Data in Mental Health: Qualitative Study of the Physicians’ Perspective in Germany
Source: J Med Internet Res. 2025 Dec 22;27:e72893. doi: 10.2196/72893 (PMC12721489; doi:10.2196/72893)
Supplement: Multimedia Appendix 2 [file jmir-v27-e72893-s002.docx]

**Prototype ESM Data Visualizations Shown to Clinicians as Discussion Stimuli**

**
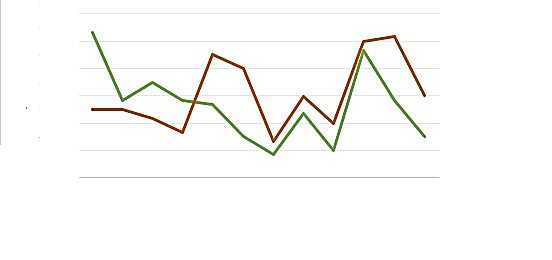
**

**Figure 1**. Prototype 1: line graph — description: for each therapy goal, patients rate their perceived progress that day and satisfaction with the progress. These data are visualized in a line chart in which a trend line can be inserted.


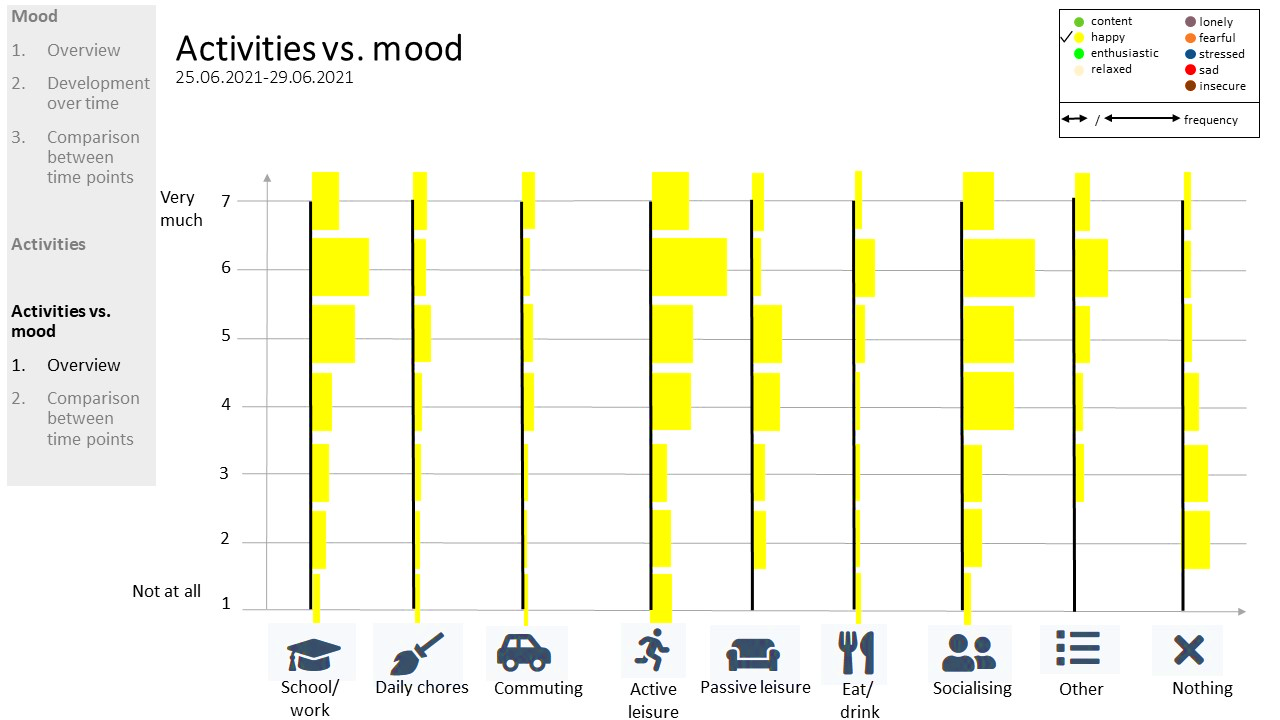


**Figure 2.** Prototype 2: activities in the context of mood —description: an overview of different activities (Item: “What are you doing right now?”) and corresponding mood rated on a likert scale (from 1 to 7). Frequency and changes in correlations are depicted over the chosen timeframe.


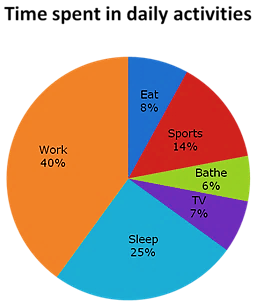


**Figure 3.** Prototype 3: pie chart —description: an overview of the patient’s daily activities can be obtained from the activity pie chart, summarizing and displaying the distribution of the different reported activities. Users can choose the timeframe for which they want to have data displayed (eg, last 7 d).
